# Successful Open Thoracoabdominal Aortic Repair in a Patient with Severe Aorto-Iliac Occlusive Disease: A Rare Case Report

**DOI:** 10.3400/avd.cr.22-00003

**Published:** 2022-06-25

**Authors:** Takanori Tsujimoto, Tatsuro Asada, Akitoshi Yamada, Kunio Gan

**Affiliations:** 1 Department of Cardiovascular Surgery, Kitaharima Medical Center, Ono, Hyogo, Japan

**Keywords:** thoracoabdominal aortic aneurysm, aorto-iliac occlusive disease, spinal cord injury

## Abstract

Open repair of thoracoabdominal aortic aneurysm (TAAA) in a patient with severe aorto-iliac occlusive disease is considered to cause an extremely high risk for spinal cord injury. A 71-year-old man who had previously undergone axillo-bifemoral bypass for aorto-iliac occlusive disease presented with persistent dilation of a TAAA. Using distal perfusion via partial extracorporeal circulation at mild hypothermia, we performed segmental sequential repair of Crawford type II TAAA. Various efforts were made to avoid spinal cord injury and ischemic visceral organ damage. Consequently, the patient completely recovered without any serious complications.

## Introduction

Open surgical repair of thoracoabdominal aortic aneurysm (TAAA) is one of the most challenging cardiovascular procedures as it continues to be associated with high morbidity and mortality.^1,2)^ Among serious complications after repair of TAAA, spinal cord injury (SCI) is one of the most devastating. A great number of factors have been demonstrated to be associated with the development of SCI after the operation, including coronary artery disease, renal insufficiency, and the extent of TAAA repair in the severely atherosclerotic aorta.^3–5)^ Particularly, TAAA patients with severe aorto-iliac occlusive disease (AIOD) are at high risk of paraplegia^6)^ because of occlusion of the infrarenal abdominal aorta and its branches, including the lumbar and internal iliac arteries, which play an important role in distal spinal cord perfusion.^7)^ We report the case of a patient with AIOD who successfully underwent surgery for TAAA without SCI, as well as strategies toward avoiding SCI and ischemic visceral organ damage during the operation.

## Case Report

**Figure 1:**
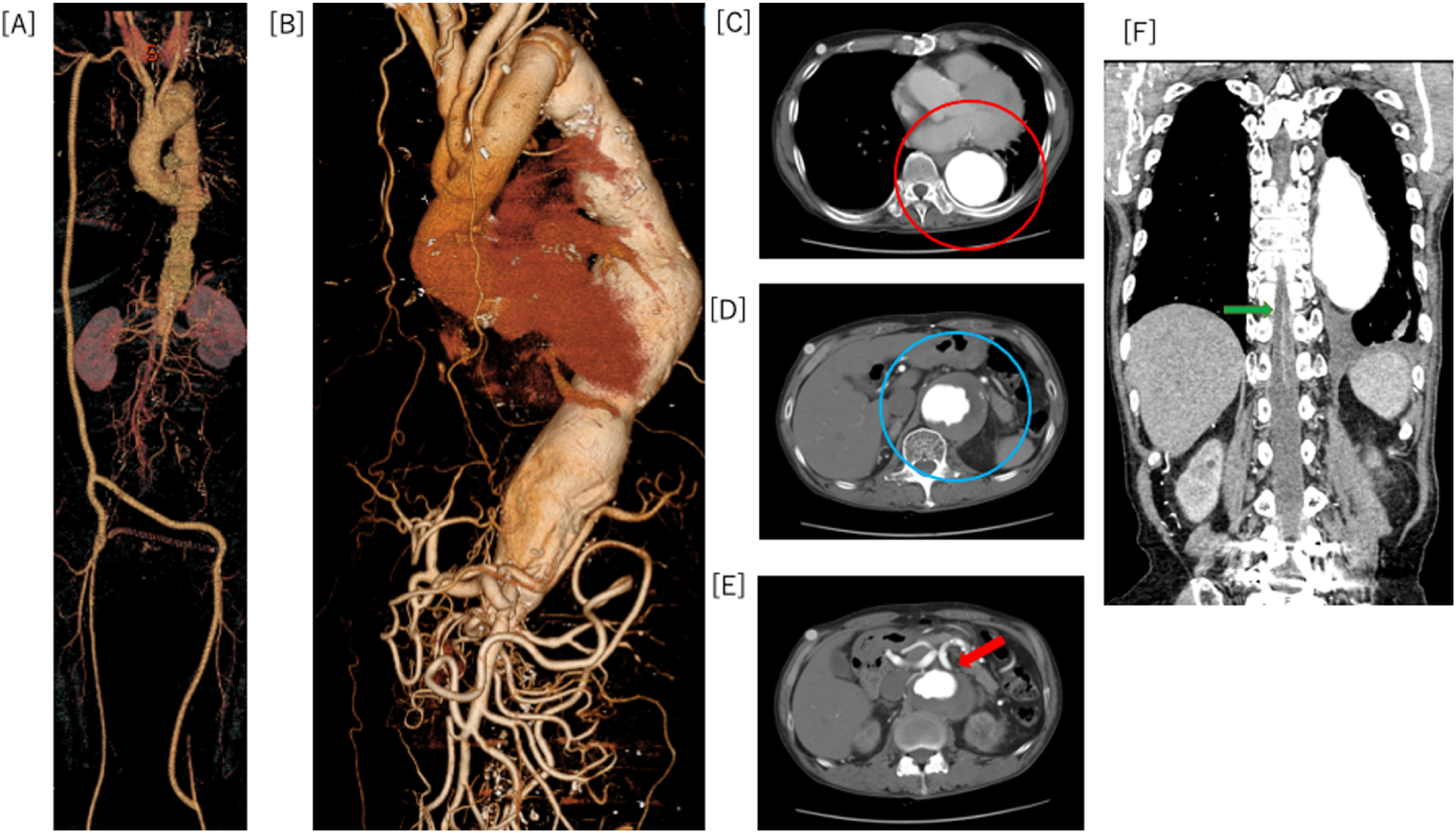
Fig. 1 Preoperative CT images.

A 71-year-old man with AIOD underwent aorto-bifemoral bypass 12 years before presentation. Nine years earlier, the left leg of this graft was occluded, and a femoro-femoral (F-F) bypass was performed. Six years earlier, the graft reoccluded. Thus, axillo-bifemoral (Ax-biF) bypass and left femoropopliteal bypass were performed, and these grafts have remained patent to date. Five years ago, the patient underwent a total arch replacement for a distal true arch aneurysm (maximum diameter of 60 mm) using the elephant trunk technique, along with coronary artery bypass grafting of the right posterior descending artery (**Fig. 1A**). After this surgery, a computed tomography (CT) scan revealed persistent dilation of a descending thoracic aortic aneurysm (DTAA) and a TAAA up to 62 and 64 mm, respectively (**Figs. 1C** and **1D**). The TAAA contained a severe mural thrombus, occluded just under the renal arteries, and the celiac artery was occluded at its origin because of median arcuate ligament syndrome (**Figs. 1B** and **1E**). On CT, the branching level of the Adamkiewicz artery (AKA) seemed to be located at the left 11th intercostal artery (ICA) (**Fig. 1F**). The patient had a medical history of myocardial infarction and cerebral infarction, complicating the past total arch replacement. Additionally, he had chronic kidney disease (serum creatine=3.12 mg/dL).

(**A**) A 3D CT image after total arch replacement. (**B**) A 3D CT image before the TAAA repair. (**C**) Axial CT image of DTAA (62 mm), red circle: DTAA. (**D**) Axial CT image of the TAAA (64 mm), blue circle: the TAAA. (**E**) Axial CT image at the CA (occluded) level; red arrow: origin of the CA. (**F**) Coronal CT image showing AKA; green arrow: AKA. CT: computed tomography; 3D: 3-dimensional; DTAA: descending thoracic aortic aneurysm; TAAA: thoracoabdominal aortic aneurysm; CA: celiac artery; AKA: Adamkiewicz artery

The day before the operation, a continuous spinal fluid drainage catheter was inserted in the lumbar region. The patient was intubated with a double-lumen endotracheal tube. Transcranial stimulated motor-evoked potentials (MEPs) were recorded throughout the operation.

**Figure 2:**
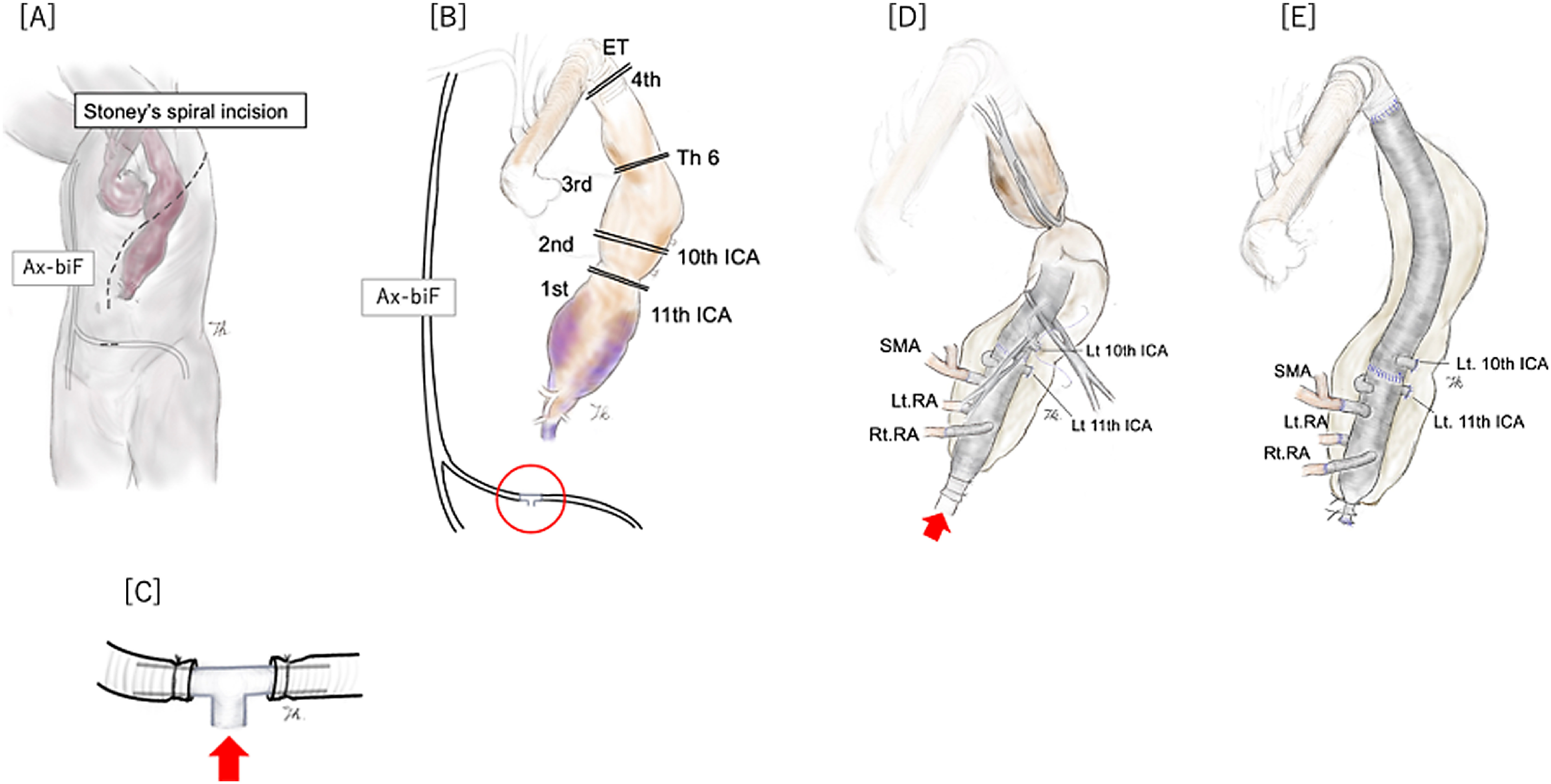
Fig. 2 Schematic representation of TAAA repair.

The entire thoracoabdominal aorta was exposed via the left fourth intercostal space, and a retroperitoneal approach was undertaken (**Fig. 2A**). After heparin sodium was administered, the left 11th ICA was exposed and temporally clamped from outside the aorta, but the MEP signals did not decrease. After clamping the left 10th ICA, the MEP signals decreased. After declamping both ICAs, the MEP signals recovered. We then decided to reconstruct both the 10th and 11th ICAs. The aorta was scanned by direct epiaortic ultrasound to examine whether aortic cross-clamping could be safely performed without embolization of the atheromatous debris.

(**A**) Skin incision (Stoney spiral incision). (**B**) Clamp sites during surgery; red circle: cannulation site. (**C**) Y-connector cannulation using an Ax-biF bypass graft. (**D**) The Lt. 10th ICA reconstruction. (**E**) Complete reconstruction of all branches. Ax-biF: axillo-bifemoral; ET: elephant trunk; ICA: intercostal artery; SMA: superior mesenteric artery; RA: renal artery; Lt: left; Rt: right

Central venous cannulation was performed using a wire-directed approach from the right femoral vein. Arterial cannulation could not be established from the femoral artery because of AIOD; thus, we exposed the F-F portion of the Ax-biF bypass graft above the pubic tubercle (**Fig. 2B**). After transection of this graft, we inserted a Y-connector to both the cutting edges of the graft and used the remaining site of the Y-connector as the arterial return cannula (**Fig. 2C**).

In this case, we initially performed distal aortic graft anastomosis during segmental aortic cross-clamping to maintain sufficient blood flow, especially to the kidney and spinal cord, using distal perfusion via partial extracorporeal circulation (p-ECC), moderate systemic heparinization (1.0 mg/kg), and mild permissive hypothermia (32°C–34°C, tympanic membrane temperature, 33°C–35°C, rectal temperature). After p-ECC was started, the descending aorta was clamped at the level of the 11th ICA, and the suprarenal portion of the abdominal aorta was opened. The celiac artery was occluded; thus, both the renal arteries (RA) and superior mesenteric artery (SMA) were perfused using 8-Fr balloon-tipped catheters via a separate single roller pump at the arterial flow. Before these procedures, we connected one of the divided arterial cannulas directly to the distal side of a 22 mm Gelweave Coselli thoracoabdominal graft with four 8 mm diameter side branches (Vascutec, Terumo, Tokyo, Japan) using an I-shaped connector to perfuse the visceral artery quickly after each anastomosis. The right RA, SMA, and left RA were anastomosed one by one to the side branches of the Coselli graft with 5-0 polypropylene, using the Carrel patch method. Then, the aortic cross-clamp site was changed just below the 10th ICA, and the origin of the 11th ICA was anastomosed to the remaining branch of the Coselli graft using the inclusion technique. To reconstruct the remaining descending aorta and the 10th ICA, a 22 mm woven J-graft Shield neo with one 9 mm branch (Japan Lifeline, Tokyo, Japan) was anastomosed to the proximal end of the Coselli graft. Aortic cross-clamping was moved to a level below the sixth ICA, the remaining aneurysm was opened, and the 10th ICA was reconstructed to the side branch of the J-graft. During this anastomosis, the 10th ICA was perfused directly through the SPF catheter, using a silicone balloon (Fuji System Corporation, Tokyo, Japan) with the inner pressure of the coronary perfusion circuit of 300 mmHg (**Fig. 2D**). Finally, the aorta was clamped above the previously implanted elephant trunk graft, and the proximal anastomosis between the elephant trunk graft and the J-graft was completed (**Fig. 2E**). Four segmental aortic cross-clamping procedures were performed before whole graft replacement (**Fig. 2B**). After the ECC, the cannulation sites of the FF portion were reconstructed, and the distal edge of the Coselli graft was closed. The ECC time was 186 min, and the operation time was 11 h and 31 min. The MEP signal did not decrease, except for the ICA clamp test.

After the operation, the patient was admitted to the intensive care unit. He regained consciousness 4 h after surgery, and movements of the lower extremities were confirmed. He was extubated 15 h after the operation. His mean blood pressure was maintained above 80 mmHg for 3 days after the operation, and the cerebrospinal fluid drainage tube was removed on the third day. He was moved to the general ward on the fourth day and was discharged 57 days after the operation. His serum creatinine level was the highest (4.05 mg/dL) on the third postoperative day and decreased gradually to 2.50 mg/dL at discharge. Hemodiafiltration was not performed after the surgery.

## Discussion

To avoid SCI, various efforts have been made routinely in our hospital, even as per previous studies.^3–5)^ It is useful to know the precise level of the origin of the AKA before reconstruction of the intercostal arteries. We determined AKA levels via preoperative enhanced CT and intraoperative clamp test of the intercostal arteries with MEP monitoring. The cerebrospinal fluid drainage tube was inserted the day before the operation. Postoperatively, hemoglobin was increased above 10 g/dL by blood transfusion, and mean blood pressure was maintained above 80 mmHg for 3 days.

Kouchoukos and colleagues^2)^ reported that an open proximal anastomosis under deep hypothermic circulation arrest (DHCA) might offer superior spinal cord protection to the arch clamping technique during repair of DTAA/TAAAs involving the distal arch. However, the routine use of DHCA for DTAA/TAAA repairs is controversial because of cerebral ischemia due to hypotension during the cooling and rewarming periods and because of the limitation of myocardial protection, which causes postoperative low cardiac output syndrome.^8)^ Moreover, because DHCA requires a longer cardiopulmonary bypass time, it causes lung dysfunction after the operation and severe coagulopathy, which may increase the risk of serious surgical bleeding.^9,10)^ When aortic cross-clamping can be safely performed via preoperative enhanced CT and intraoperative direct epiaortic ultrasound, we prefer a physiologic arch clamping technique using p-ECC with a beating heart at mild hypothermia.

TAAA repair procedures for AIOD patients are not only at high risk of paraplegia due to the lack of distal spinal cord perfusion but they are also exposed to the highly complicated procedure of establishing p-ECC. In this situation, maintaining the flow balance between venous drainage and arterial reinfusion by femoral return cannulation is challenging. Severe general hypotension is easily induced, especially during the starting period of p-ECC and massive intraoperative bleeding. In this case, the preexisting Ax-biF bypass was patent, and we used this graft as an arterial return cannula (**Figs. 2B** and **2C**). This method is effective not only toward obtain stable hemodynamics throughout the operation but also toward obtaining sufficient blood flow to the lower extremities during aortic cross-clamping.

In this case, we expected that we could not obtain sufficient blood flow to the visceral organs from the collateral channels during distal perfusion from the p-ECC during aortic cross-clamping. To reduce the ischemic time of the visceral organs, we performed distal anastomosis first (**Fig. 2B**). Using the segmental aortic cross-clamping technique, we made the first clamping below the level of the 11th intercostal space, opened the distal suprarenal abdominal aorta, perfused the separate visceral organs an 8-Fr cannula with a balloon, and reconstructed the visceral vessels as soon as it was feasible. After each visceral anastomosis was accomplished, perfusion was determined from direct return cannulation of the distal end of the Coselli graft. During this period, the AKA blood flow was maintained by the beating proximal normo-pressure blood flow.

## Conclusion

A 71-year-old high-risk patient with a history of Ax-biF bypass for AIOD successfully underwent operative graft replacement for an extended type II TAAA. We described very effective techniques to prevent ischemia of the visceral organs and spinal cord that were used in this case.
